# A *De Novo* Mutation in *DYRK1A* Causes Syndromic Intellectual Disability: A Chinese Case Report

**DOI:** 10.3389/fgene.2019.01194

**Published:** 2019-11-19

**Authors:** Fengchang Qiao, Binbin Shao, Chen Wang, Yan Wang, Ran Zhou, Gang Liu, Lulu Meng, Ping Hu, Zhengfeng Xu

**Affiliations:** Department of Prenatal Diagnosis, Women’s Hospital of Nanjing Medical University, Nanjing Maternity and Child Health Care Hospital, Nanjing, China

**Keywords:** DYRK1A, intellectual disability, microcephaly, nonsense mutation, whole-exome sequencing

## Abstract

Autosomal dominant mental retardation-7 (MRD7) is a rare anomaly, characterized by severe intellectual disability, feeding difficulties, behavior abnormalities, and distinctive facial features, including microcephaly, deep-set eyes, large simple ears, and a pointed or bulbous nasal tip. Some studies show that the disorder has a close correlation with variants in *DYRK1A*. Herein we described a Chinese girl presenting typical clinical features diagnosed at 4 years old. Whole-exome sequencing of the familial genomic DNA identified a novel mutation c.930C > A (p.Tyr310*) in exon 7 of *DYRK1A* in the proband. The nonsense mutation was predicted to render the truncation of the protein. Our results suggested that the *de novo* heterozygous mutation in *DYRK1A* was responsible for the MRD7 in this Chinese family, which both extended the knowledge of mutation spectrum in MRD7 patients and highlighted the clinical application of exome sequencing.

## Background

*DYRK1A* encodes dual specificity tyrosine-phosphorylation-regulated kinase 1A, which contains a nuclear targeting signal sequence, a protein kinase domain, a leucine zipper motif, and a highly conserved 13-consecutive-histidine repeat. This protein catalyzes the phosphorylation of serine and threonine residues on exogenous substrates, as well as phosphorylation of its own kinase domain. The protein is ubiquitously expressed in fetal and adult tissues, with a high expression in the brain (Song et al., 1996; Galceran et al., 2003; Martinez de Lagran et al., 2012). DYRK1A-related intellectual disability syndrome is characterized by mild to severe range of intellectual disability including impaired speech development, microcephaly, and autism spectrum disorder including anxious and/or stereotypic behavior problems. Also, affected individuals often have other symptoms such as typical facial gestalt, feeding problems, seizures, hypertonia, gait disturbances, and skeletal system abnormalities. Rarely, endocrine problems and dental, ophthalmologic, and/or cardiac anomalies are reported (Bronicki et al., 2015; Ji et al., 2015; Ruaud et al., 2015; van Bon et al., 2016). To date, there are more than 2,000 genes associated with intellectual disability (Human Phenotype Ontology Database: http://compbio.charite.de/hpoweb/showterm?id=HP:0000118#id=HP:0001249). Deciphering developmental disorders study group and studies showed that *DYRK1A*-related intellectual disability syndrome accounted for 0.1–0.5% of individuals with intellectual disability and/or autism (Courcet et al., 2012; O’Roak et al., 2012; van Bon et al., 2016).

As the technologies develop, DYRK1A-related intellectual disability syndrome was detected by cytogenetic analysis and Fluorescence in situ hybridization (FISH) to investigate karyotype abnormalities, chromosomal microarray analysis (CMA) or array-CGH to find copy number variations (CNV), and direct Polymerase Chain Reaction (PCR)/panel/clinical exome sequencing/exome sequencing to explore single nucleotide variants and small insertions/deletions (Moller et al., 2008; Yamamoto et al., 2011; Courcet et al., 2012; Valetto et al., 2012; Redin et al., 2014; Bronicki et al., 2015; Ji et al., 2015; Rump et al., 2016; van Bon et al., 2016; Weisfeld-Adams et al., 2016; Evers et al., 2017).

In this study, we identify a novel nonsense mutation in *DYRK1A* by whole-exome sequencing (WES) in a small Chinese family with the severe phenotype of the syndrome. We also review the previously published cases originated from the ClinVar database and Medline search to further summarize variations in *DYRK1A* identified in MRD7, which provide convenience for clinical application.

## Materials and Methods

### Patient Samples

The patient with clinical features was highly consistent with MRD7. Parents took her to Nanjing Maternity and Child Health Care Hospital for genetic counseling when she was 4 years old. Written informed consent form was obtained from the legal guardians of the patient for the molecular genetic analysis and the publication of this case report. Our study was approved by the Ethics Committee of the hospital. Peripheral blood of the family was collected in EDTA anticoagulant tubes, and genomic DNA was isolated from 400 μl peripheral blood using the Automated Nucleic Acid Extractor (Concert Bioscience, Xiamen, China) according to the manufacturer’s protocols.

### Chromosomal Microarray Analysis

Human cyto12 single-nucleotide polymorphism (SNP) array beadchip with 300,000 probes (Illumina, San Diego, CA, USA) (Leung et al., 2011) was used for whole genome scan. SNP array was carried out according to the manufacturer’s instructions. For SNP array, CNV analysis was carried out using KaryoStudio V1.4.3.0 (Hu et al., 2015).

### Whole-Exome Sequencing

DNA of the trio was sent for whole exome capture using the SureSelect Human All Exon V6 (Agilent Technologies, USA). According to the manufacturer’s recommendations, the resulting libraries was sequenced on Illumina HiSeq 2000. Over 85 Mbs of mappable sequences were generated, resulting in a depth of coverage﹥30X for more than 97.4% of RefSeq-coding exons. Reads were aligned to Human genome GRCh37/hg19 with the Burrows-Wheeler Aligner36 (BWA.0.6.2) (Li and Durbin, 2010). The Genome Analysis Toolkit 2.6-4 (McKenna et al., 2010) was used for genotyping and indel discovery and single-nucleotide variants, as well as for indel realignment and base quality score recalibration. Candidate events were inspected using the Integrative Genomics Viewer (IGV), while coverage was evaluated with the GATK Depth of Coverage tool by rejecting bases with base quality of <30 and reads with mapping quality of <20.

### Sanger Sequencing

PCR primers were designed to amplify exon 7 of *DYRK1A*. The mutation of the proband was sequenced from a 230 bp DNA fragment amplified using primer pair 5’-TGTTGAAGTTAATCAATGGAACCCT-3’ and 5’-ACCCGAGGGACCACATA TCA-3’. Sanger sequencing was performed using the ABI 3730xl DNA automated sequencer (Applied Biosystems, Foster City, CA, USA).

### Case Presentation

The girl was the firstborn child of healthy nonconsanguineous Chinese parents from Anhui Province. She was cesarean born with a length of 47 cm (−2 SD), weight of 3180 g (−1 SD), and a head circumference of 31 cm (<−2 SD). From birth onward, she had feeding problems and febrile seizures, regularly. The electroencephalogram (EEG) was normal or slightly abnormal during the first year of life. She was able to sit unsupported around the age of 10 months and walk independently around the age of 23 months, and non-fluent motoric movements and hypoactivity were noted. She was described as having a broad-based clumsy tread, and exhibited a mild tremor. She started to pronounce syllables around 2 years old and used simple words around the age of 3 years. Nowadays, she could not speak with full sentences and presented with significantly lower IQ (67).

**Figure 1:**
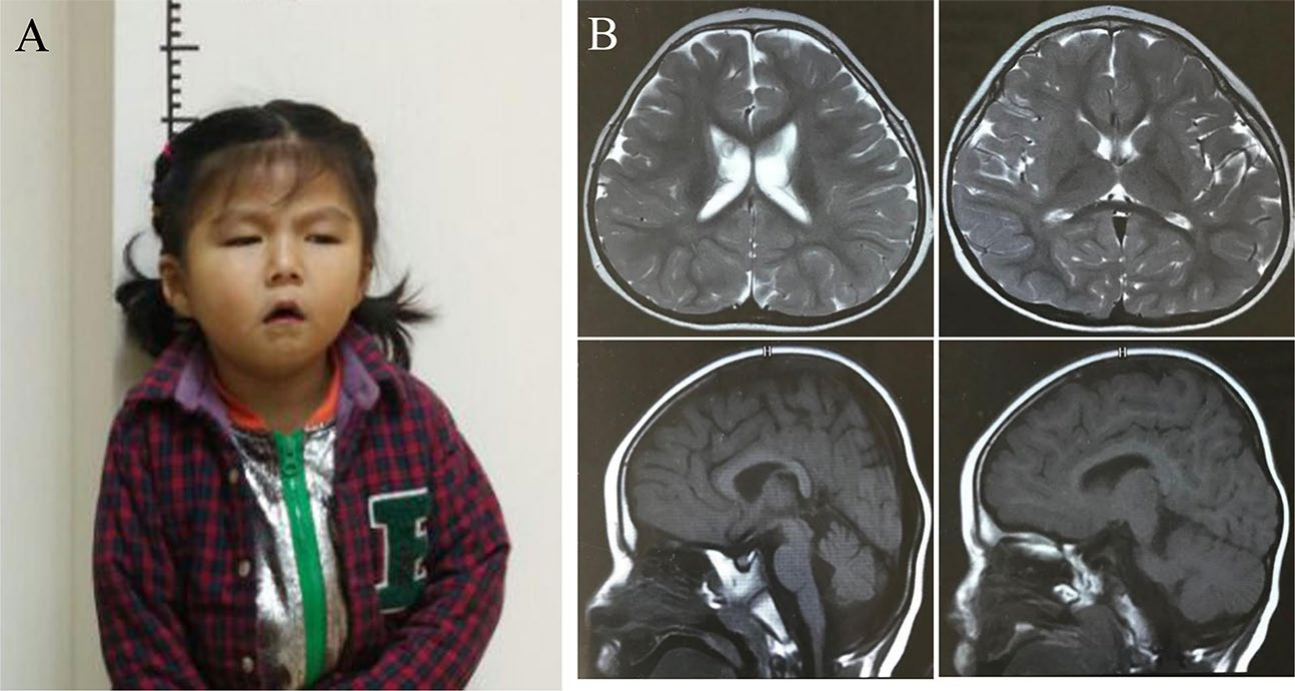
Clinical and imaging features of the proband **(A)** The picture illustrates the facial dysmorphic features consisting of microcephaly, micrognathia, deep-set eyes, large ears, pointed nasal tip, and a downturned mouth. Written informed consent for publication of medical data and identifiable images was obtained from the parents of the patient. **(B)**. Brain MR images showed mild prominence of lateral ventricles, enlarged pericerebral spaces, high palate, delayed myelination, and a thin corpus callosum when the girl was at 4 years of life.

At 4 years old, the girl had a slender posture with a length of 90 cm (−1.5 SD) and presented with significantly smaller head size of 38.5 cm (<−2 SD) compared to the same age children. Typical facial dysmorphisms included deep-set eyes, pointed nasal tip, large ears, a downturned mouth, and micrognathia (Figure 1A). A cerebral magnetic resonance imaging (MRI) showed mild widened lateral ventricles, enlarged pericerebral spaces, high palate, a thin corpus callosum, and delayed myelination but without structural congenital anomalies (Figure 1B).

**Figure 2:**
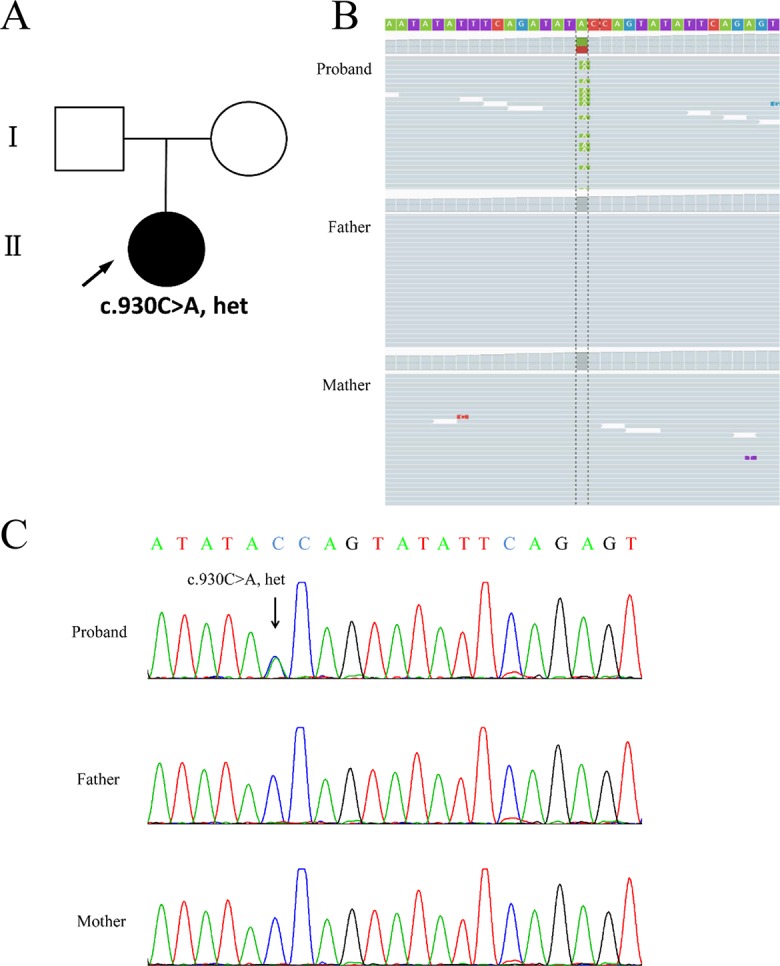
Genetic findings from the family. **(A)** Pedigree of the family with segregation of the identified *DYRK1A* mutation. The square represents male, and circles represent female. Filled symbol indicates the affected individual. **(B)** Visualization of mutation in the *DYRK1A* gene is shown on the Integrative Genomics Viewer. Variation c.930C > A was heterozygous in the proband. **(C)** The variation of c.930C > A is a nonsense mutation (p.Tyr310*) identified in the proband. The parents were tested and did not carry the mutation. Black arrows indicate the point mutation.

Chromosomal abnormalities and submicroscopic chromosomal imbalances at the whole genome level of the girl by CMA did not reveal any anomaly (data not shown). Using WES, a heterozygous nonsense variant (chr21:38865324C > A; c.930C > A; p.Tyr310*) in the coding region of exon 7 of the *DYRK1A* gene (NM_130436.2) was identified in the proband. Nevertheless, none of the mutation at this site was found in her parents (Figures 2A, B). Sanger sequencing confirmed this conclusion (Figure 2C).

The nonsense variant in the amino acid residue tyrosine 310, a perfectly conserved amino acid in *DYRK1A* and *DYRK1A* vertebrate orthologues, was a heterozygous truncation mutation and interfered with the protein kinase activity. This variant was absent from the Genome Aggregation Database browsers (gnomAD, http://gnomad.broadinstitute.org/) and the Exome Aggregation Consortium databases (ExAC, http://exac.broadinstitute.org/) or dbSNP (http://www.ncbi.nlm.nih.gov/snp), which excluded that it represents a polymorphism. Meanwhile, the loss-of-function intolerance (pLI) for *DYRK1A* is 1.00, which is based on the ExAC sequencing data, suggesting strong intolerance to functional mutations. Taken together, we hypothesize that the nonsense mutation causes the severe clinical phenotype by ACMG recommendations for standards and guidelines for the interpretation of sequence variants (Richards et al., 2015). A heterozygous variant in PKHD1L1 (NM_177531.6: c.9352G>T, Glu3118*); a heterozygous variant in PRODH2 (NM_021232.1: c.457C>T, Arg153*) and a heterozygous variant in SDK2 (NM_001144952.2: c.1865delT, Leu622Argfs*29) was also identified, which were ruled out because the genes were recessive inheritance and only one variant of each gene was identified in the patient. Moreover, the phenotype of the patient was not consistent with the genes (**Supplementary Table S1**).

## Discussion

In this study, we described a Chinese girl characterized by severe intellectual disability, delayed motor development, seizures, microcephaly, and special facial features for the first time. Using WES, we identified a *de novo* heterozygous truncated mutation (c. 930C > A, p.Tyr310*) in *DYRK1A*, which was responsible for autosomal dominant mental retardation-7 (MRD7). This mutation was predicted to cause premature truncation of the DYRK1A protein and presumably impairment of kinase activity, thereby contributing to phenotype in the patient. The highly conserved dual-specificity tyrosine-phosphorylation-regulated kinase (DYRK) family includes the members of *DYRK1B*, *DYRK2*, *DYRK3*, and *DYRK4*. As a member of the DYRK subfamily, *DYRK1A* contains a kinase domain, located centrally in the protein, spanning from amino acid residue 158 to residue 479. Meanwhile, *DYRK1A* comprises two nuclear localization signals at the N-terminus (NLS) (Soundararajan et al., 2013), a DYRK homology (DH)-box, a PEST domain, a speckle-targeting signal (STS), a histidine repeat, and a region rich in serines and threonines at the C-terminus (Song et al., 1996). During the early embryonic development in Drosophila, *DYRK1A* is expressed in neuroepithelial progenitor cells defined the transition step from proliferations to neurogenic divisions (Hammerle et al., 2002). Remarkably, mice with heterozygous mutation of *DYRK1A* showed a significant body size reduction and a decreased size of the brain than those wild type mice, and mice with one functional copy of the *DYRK1A* gene also revealed motor defects, altered behaviors, and intrauterine growth restriction (Fotaki et al., 2002; Fotaki et al., 2004; Martinez de Lagran et al., 2007). Tejedor et al. reviewed the role of *DYRK1A* gene in neurogenesis and characterized the protein as a regulator of multiple neurodevelopmental procedures, itemizing many possible interacting proteins and/or substrates (Tejedor and Hammerle, 2011).

In human, the *DYRK1A* gene located in 21q22.2 on chromosome 21 maps to the critical region of Down syndrome (DS) (Guimera et al., 1996). In 2008, Moller et al. firstly described two unrelated patients with truncation of *DYRK1A* lead to microcephaly, developmental delay, feeding difficulty, and epilepsy, probably through restrain neural differentiation (Moller et al., 2008). Courcet et al. identified a 69 kb deletion and a frameshift mutation (c.290_291delCT; p.Ser97Cysfs*98) in *DYRK1A*. While microcephaly, language delay, and seizures were considered as fixed features, *DYRK1A* mutation was found in 1/70 patients (1.4%). Hence, the authors suggested that *DYRK1A* analysis could be mostly considered when patients present with the phenotype (Courcet et al., 2012).

The strikingly similar features of previously reported individuals with MRD7 were intrauterine growth retardation (IUGR), microcephaly, severe intellectual disability, brain abnormalities (MRI), global developmental delay, speech and motor delay, seizures, behavioral issues, feeding difficulties, broad-based gait, and dysmorphic facies (Bronicki et al., 2015; Luco et al., 2016; Widowati et al., 2018). In this report, the patient with the *de novo* heterozygous mutation of *DYRK1A* (c.930C > A, p.Tyr310*) displayed typically clinical features, which are closely resembling the syndrome.

**Figure 3:**
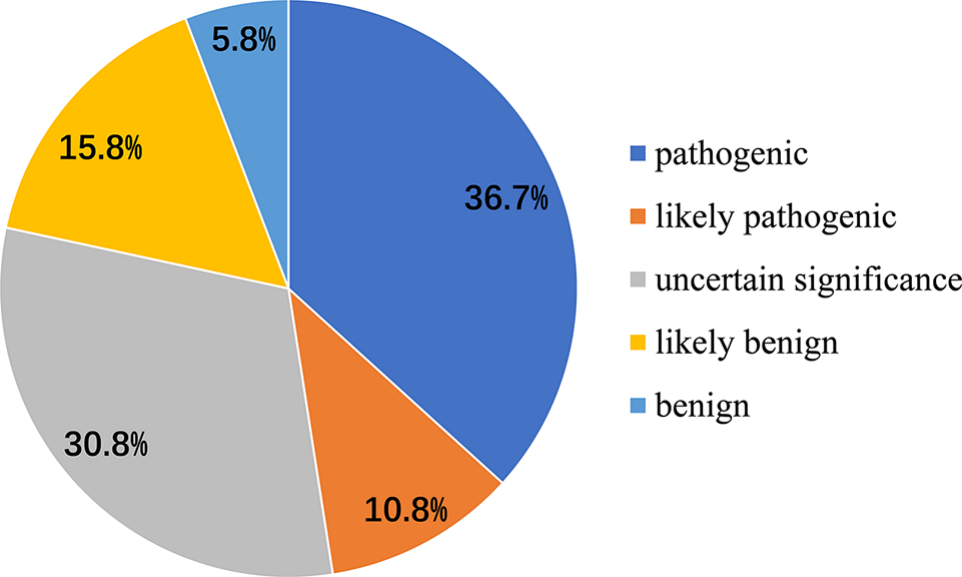
Classification of mutations in *DYRK1A* associated with MRD7 Pathogenic variants were seen in 36.7%, likely pathogenic variants were 10.8%, benign variants were found in 5.8%, and likely benign variants and uncertain significance variants were 37/120 (30.8%) and 19/120 (15.8%), respectively.

**Figure 4:**
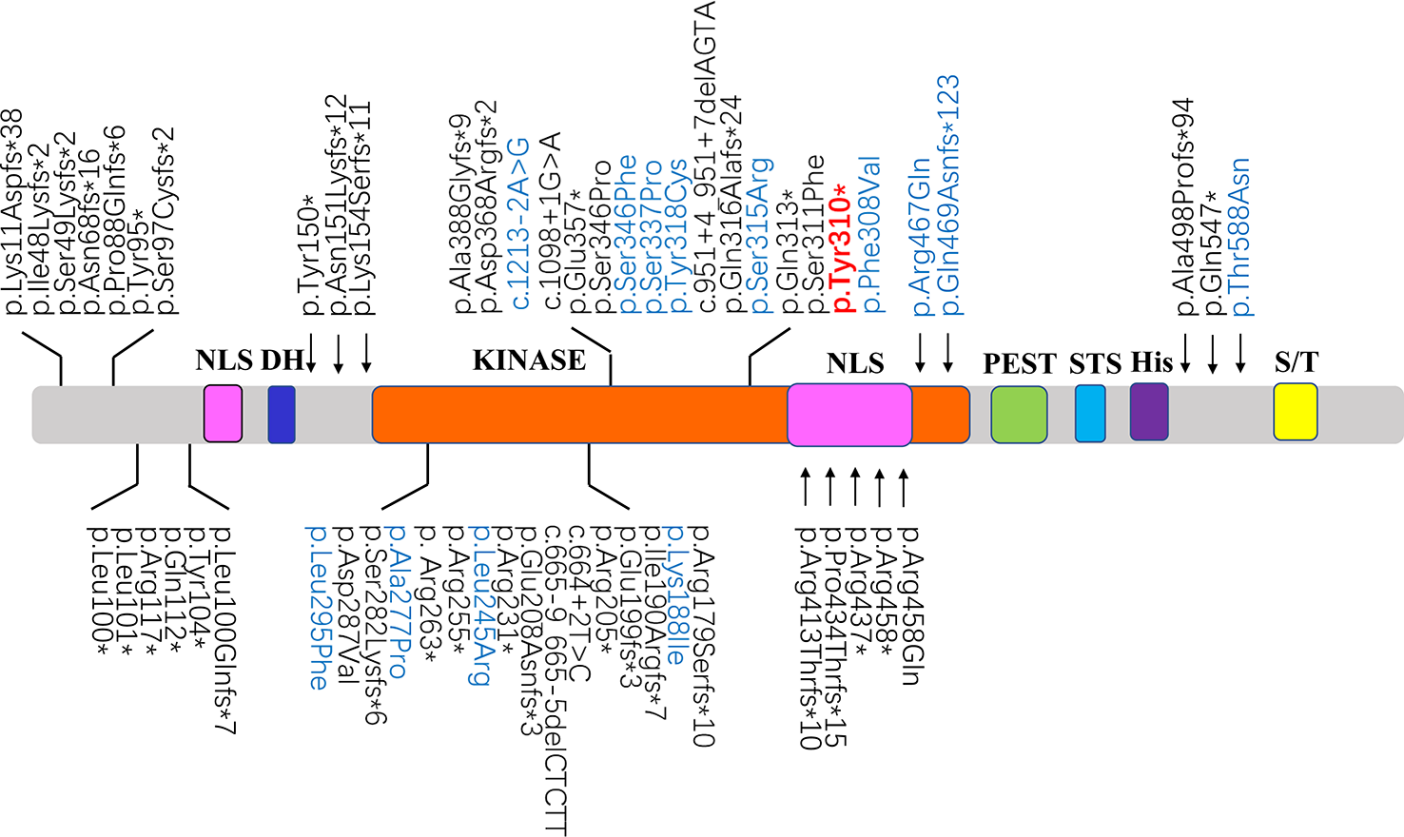
Schematic diagrams showing structure of DYRK1A. The recently identified pathogenic variants and likely pathogenic variants were shown in black and blue, respectively. The position of the mutation identified in this study are shown in red. NLS, nuclear localization signal; DH, DYRK homology domain; KINASE, protein kinase domain; PEST, domain enriched in proline, glutamic acid, serine, and threonine residues; STS, the speckle-targeting signal; His, histidine repeats; S/T, serine and threonine-rich region.

We also analyzed 120 disease-associated variants obtained from the ClinVar database and the formerly reported Medline search (**Supplementary Table S2**). These reported variants were all absent from the parental genomes, thereby proving their *de novo* occurrence. Uncertain significance variants and likely benign variants were found in 37/120 (30.8%) and 19/120 (15.8%). Benign variants were observed in 7/120 (5.8%). Remarkable, 36.7% was recognized as pathogenic variants (44/120) likely pathogenic variants were seen in 13/120 (10.8%) (**Figure 3**). Among the pathogenic and likely pathogenic variants(**Figure 4**), the disruptive mutations included nonsense, frameshift, and splice site variants, which affected residues widespread within the protein, all predicted premature stop codons leading to a potential loss of function.

Technological improvement in high-throughput sequencing has satisfied clinical requirements and thus hastened the identification of novel pathological variants in *DYRK1A* gene (Yamamoto et al., 2011; Ji et al., 2015; Ruaud et al., 2015; van Bon et al., 2016). As compared to conventional sanger sequencing, WES provides an effective alternative method that aids genetic diagnosis especially in cases with overlapping features among neurogenesis. Additionally, the clinical utility of WES would be promoted significantly within the near future as the cost is reduced.

### Concluding Remarks

In conclusion, we identified a novel nonsense mutation in *DYRK1A* in a Chinese family, expanding the mutation spectrum of MRD7. Meanwhile, we provide a review of the formerly reported cases to summarize variations in *DYRK1A* gene, which can provide convenience for clinical application. And also, we emphasize the value of WES for genetic diagnosis in rare diseases.

## Data Availability Statement

All datasets for this study are included in the article/**Supplementary Material**.

## Ethics Statement

The study was reviewed and approved by the Ethics Committee of Nanjing Maternity and Child Health Care Hospital in China. The parents of the patient provided written informed consent for publication of medical data and for molecular genetic analysis of the related gene.

## Author Contributions

FQ and BS performed the experiments, interpreted the data, and wrote the manuscript. CW, YW, and RZ collected clinical information and provided genetics counseling. GL provided patient samples and determined the phenotype based on the clinical criteria. LM performed experiments and analyzed the data. PH and ZX designed the study and revised the manuscript.

## Funding

This work was supported by the National Natural Science Foundation of China (81602300, 81770236, 81801445, 81701427), National Key R&D Program of China (2018YFC1002402), Natural Science Foundation of Jiangsu Province (BK20160139, BK20181121), and Project on Maternal and Child Health Talents of Jiangsu Province (FRC201791).

## Conflict of Interest

The authors declare that the research was conducted in the absence of any commercial or financial relationships that could be construed as a potential conflict of interest.

## Abbreviations

MRD7, Autosomal Dominant Mental Retardation-7 tal Retardation-7; DYRK, Dual-Specificity Tyrosine Phosphorylation-Regulated Kinase; DYRK1A, Dual-Specificity Tyrosine Phosphorylation-Regulated Kinase 1A; SNP, Single-Nucleotide Polymorphism; CNV, Copy Number Variation; MRI, Magnetic Resonance Imaging; WES, Whole Exome Sequencing.

## References

[B1] Bronicki L. M. Redin C. Drunat S. Piton A. Lyons M. Passemard S. (2015). Ten new cases further delineate the syndromic intellectual disability phenotype caused by mutations in DYRK1A. Eur. J. Hum. Genet. 23 (11), 1482–1487. 10.1038/ejhg.2015.29 25920557 PMC4613470

[B2] Courcet J. B. Faivre L. Malzac P. Masurel-Paulet A. Lopez E. Callier P. (2012). The DYRK1A gene is a cause of syndromic intellectual disability with severe microcephaly and epilepsy. J. Med. Genet. 49 (12), 731–736. 10.1136/jmedgenet-2012-101251 23099646

[B3] Evers J. M. Laskowski R. A. Bertolli M. Clayton-Smith J. Deshpande C. Eason J. (2017). Structural analysis of pathogenic mutations in the DYRK1A gene in patients with developmental disorders. Hum. Mol. Genet. 26 (3), 519–526. 10.1093/hmg/ddw409 28053047 PMC5409128

[B4] Fotaki V. Dierssen M. Alcantara S. Martinez S. Marti E. Casas C. (2002). Dyrk1A haploinsufficiency affects viability and causes developmental delay and abnormal brain morphology in mice. Mol. Cell Biol. 22 (18), 6636–6647. 10.1128/mcb.22.18.6636-6647.2002 12192061 PMC135639

[B5] Fotaki V. Martinez De Lagran M. Estivill X. Arbones M. Dierssen M. (2004). Haploinsufficiency of Dyrk1A in mice leads to specific alterations in the development and regulation of motor activity. Behav. Neurosci. 118 (4), 815–821. 10.1037/0735-7044.118.4.815 15301607

[B6] Galceran J. de Graaf K. Tejedor F. J. Becker W. (2003). The MNB/DYRK1A protein kinase: genetic and biochemical properties. J. Neural. Transm. Suppl (67), 139–148. 10.1007/978-3-7091-6721-2_12 15068246

[B7] Guimera J. Casas C. Pucharcos C. Solans A. Domenech A. Planas A. M. (1996). A human homologue of Drosophila minibrain (MNB) is expressed in the neuronal regions affected in Down syndrome and maps to the critical region. Hum. Mol. Genet. 5 (9), 1305–1310. 10.1093/hmg/5.9.1305 8872470

[B8] Hammerle B. Vera-Samper E. Speicher S. Arencibia R. Martinez S. Tejedor F. J. (2002). Mnb/Dyrk1A is transiently expressed and asymmetrically segregated in neural progenitor cells at the transition to neurogenic divisions. Dev. Biol. 246 (2), 259–273. 10.1006/dbio.2002.0675 12051815

[B9] Hu P. Meng L. Ma D. Qiao F. Wang Y. Zhou J. (2015). A novel 11p13 microdeletion encompassing PAX6 in a Chinese Han family with aniridia, ptosis and mental retardation. Mol. Cytogenet. 8 (1), 3. 10.1186/s13039-015-0110-2 25628759 PMC4307215

[B10] Ji J. Lee H. Argiropoulos B. Dorrani N. Mann J. Martinez-Agosto J. A. (2015). DYRK1A haploinsufficiency causes a new recognizable syndrome with microcephaly, intellectual disability, speech impairment, and distinct facies. Eur. J. Hum. Genet. 23 (11), 1473–1481. 10.1038/ejhg.2015.71 25944381 PMC4613469

[B11] Leung T. Y. Vogel I. Lau T. K. Chong W. Hyett J. A. Petersen O. B. (2011). Identification of submicroscopic chromosomal aberrations in fetuses with increased nuchal translucency and apparently normal karyotype. Ultrasound Obstet. Gynecol. 38 (3), 314–319. 10.1002/uog.8988 21400624

[B12] Li H. Durbin R. (2010). Fast and accurate long-read alignment with Burrows-Wheeler transform. Bioinformatics 26 (5), 589–595. 10.1093/bioinformatics/btp698 20080505 PMC2828108

[B13] Luco S. M. Pohl D. Sell E. Wagner J. D. Dyment D. A. Daoud H. (2016). Case report of novel DYRK1A mutations in 2 individuals with syndromic intellectual disability and a review of the literature. BMC Med. Genet. 17, 15. 10.1186/s12881-016-0276-4 26922654 PMC4769499

[B14] Martinez de Lagran M. Bortolozzi A. Millan O. Gispert J. D. Gonzalez J. R. Arbones M. L. (2007). Dopaminergic deficiency in mice with reduced levels of the dual-specificity tyrosine-phosphorylated and regulated kinase 1A, Dyrk1A(+/-). Genes Brain Behav. 6 (6), 569–578. 10.1111/j.1601-183X.2006.00285.x 17137466

[B15] Martinez de Lagran M. Benavides-Piccione R. Ballesteros-Yanez I. Calvo M. Morales M. Fillat C. (2012). Dyrk1A influences neuronal morphogenesis through regulation of cytoskeletal dynamics in mammalian cortical neurons. Cereb. Cortex 22 (12), 2867–2877. 10.1093/cercor/bhr362 22215728

[B16] McKenna A. Hanna M. Banks E. Sivachenko A. Cibulskis K. Kernytsky A(2010). The Genome Analysis Toolkit: a MapReduce framework for analyzing next-generation DNA sequencing data. Genome Res. 20 (9), 1297–1303. 10.1101/gr.107524.110 20644199 PMC2928508

[B17] Moller R. S. Kubart S. Hoeltzenbein M. Heye B. Vogel I. Hansen C. P. (2008). Truncation of the Down syndrome candidate gene DYRK1A in two unrelated patients with microcephaly. Am. J. Hum. Genet. 82 (5), 1165–1170. 10.1016/j.ajhg.2008.03.001 18405873 PMC2427221

[B18] O’Roak B. J. Vives L. Fu W. Egertson J. D. Stanaway I. B. Phelps I. G. (2012). Multiplex targeted sequencing identifies recurrently mutated genes in autism spectrum disorders. Science 338 (6114), 1619–1622. 10.1126/science.1227764 23160955 PMC3528801

[B19] Redin C. Gerard B. Lauer J. Herenger Y. Muller J. Quartier A. (2014). Efficient strategy for the molecular diagnosis of intellectual disability using targeted high-throughput sequencing. J. Med. Genet. 51 (11), 724–736. 10.1136/jmedgenet-2014-102554 25167861 PMC4215287

[B20] Richards S. Aziz N. Bale S. Bick D. Das S. Gastier-Foster J. et al. (2015). Standards and guidelines for the interpretation of sequence variants: a joint consensus recommendation of the American College of Medical Genetics and Genomics and the Association for Molecular Pathology. Genet Med. 17 (15), 405–424. 10.1038/gim.2015.30 25741868 PMC4544753

[B21] Ruaud L. Mignot C. Guet A. Ohl C. Nava C. Heron D. (2015). DYRK1A mutations in two unrelated patients. Eur. J. Med. Genet. 58 (3), 168–174. 10.1016/j.ejmg.2014.12.014 25641759

[B22] Rump P. Jazayeri O. van Dijk-Bos K. K. Johansson L. F. van Essen A. J. Verheij J. B. (2016). Whole-exome sequencing is a powerful approach for establishing the etiological diagnosis in patients with intellectual disability and microcephaly. BMC Med. Genomics 9, 7. 10.1186/s12920-016-0167-8 26846091 PMC4743197

[B23] Song W. J. Sternberg L. R. Kasten-Sportes C. Keuren M. L. Chung S. H. Slack A. C. (1996). Isolation of human and murine homologues of the Drosophila minibrain gene: human homologue maps to 21q22.2 in the Down syndrome “critical region”. Genomics 38 (3), 331–339. 10.1006/geno.1996.0636 8975710

[B24] Soundararajan M. Roos A. K. Savitsky P. Filippakopoulos P. Kettenbach A. N. Olsen J. V. (2013). Structures of Down syndrome kinases, DYRKs, reveal mechanisms of kinase activation and substrate recognition. Structure 21 (6), 986–996. 10.1016/j.str.2013.03.012 23665168 PMC3677093

[B25] Tejedor F. J. Hammerle B. (2011). MNB/DYRK1A as a multiple regulator of neuronal development. FEBS J. 278 (2), 223–235. 10.1111/j.1742-4658.2010.07954.x 21156027

[B26] Valetto A. Orsini A. Bertini V. Toschi B. Bonuccelli A. Simi F. (2012). Molecular cytogenetic characterization of an interstitial deletion of chromosome 21 (21q22.13q22.3) in a patient with dysmorphic features, intellectual disability and severe generalized epilepsy. Eur. J. Med. Genet. 55 (5), 362–366. 10.1016/j.ejmg.2012.03.011 22548977

[B27] van Bon B. W. Coe B. P. Bernier R. Green C. Gerdts J. Witherspoon K. (2016). Disruptive de novo mutations of DYRK1A lead to a syndromic form of autism and ID. Mol. Psychiatry 21 (1), 126–132. 10.1038/mp.2015.5 25707398 PMC4547916

[B28] Weisfeld-Adams J. D. Tkachuk A. K. Maclean K. N. Meeks N. L. Scott S. A. Luco S. M. (2016). A de novo 2.78-Mb duplication on chromosome 21q22.11 implicates candidate genes in the partial trisomy 21 phenotype Case report of novel DYRK1A mutations in 2 individuals with syndromic intellectual disability and a review of the literature. NPJ Genom. Med. 1, 15. 10.1038/npjgenmed.2016.310.1186/s12881-016-0276-4

[B29] Widowati E. W. Ernst S. Hausmann R. Muller-Newen G. Becker W. (2018). Functional characterization of DYRK1A missense variants associated with a syndromic form of intellectual deficiency and autism. Biol Open. 7 (4). 10.1242/bio.032862 PMC5936063 29700199

[B30] Yamamoto T. Shimojima K. Nishizawa T. Matsuo M. Ito M. Imai K. (2011). Clinical manifestations of the deletion of Down syndrome critical region including DYRK1A and KCNJ6 . Am. J. Med. Genet. A 155a (1), 113–119. 10.1002/ajmg.a.33735 21204217

