# Supplementary material for: A De Novo Mutation in DYRK1A Causes Syndromic Intellectual Disability: A Chinese Case Report
Source: Front Genet. 2019 Nov 19;10:1194. doi: 10.3389/fgene.2019.01194 (PMC6877748; doi:10.3389/fgene.2019.01194)
Supplement: Supplementary file 1 [file DataSheet_1.pdf]

Table S1: High priority variants in the proband from exome sequence analysis

Gene	Transcript	Nucleotide change	Amino acid change	Zygosity	Inheritance	ACMG classification
PKHD1L1	NM_177531.6	c.9352G>T	Glu3118*	Het	AR	pathogenic
PRODH2	NM_021232.1	c.457C>T	Arg153*	Het	AR	pathogenic
SDK2	NM_001144952.2	c.1865delT	Leu622Argfs*29	Het	AR	Likely pathogenic
