# Supplementary material for: A De Novo Mutation in DYRK1A Causes Syndromic Intellectual Disability: A Chinese Case Report
Source: Front Genet. 2019 Nov 19;10:1194. doi: 10.3389/fgene.2019.01194 (PMC6877748; doi:10.3389/fgene.2019.01194)
Supplement: Supplementary file 2 [file DataSheet_2.pdf]

Table S2: Overview of previously reported mutations in *DYRK1A* gene identified in Mental retardation, autosomal dominant 7.

Chromosome coordinate (GRCh37/hg19)	Transcript	Nucleotide change	Amino acid change	Clinical significance	ClinVar accession number	Reference
**Chr21:38845006**	NM_001396.3	c.31delA	p.Lys11Aspfs*38	Pathogenic		PMID:28053047
**Chr21:38845117 -38845118**	NM_001396.3	c.142_143delAT	p.Ile48Lysfs*2	Pathogenic	SCV000056590.3	PMID:23160955
**Chr21:38845118**	NM_001396.4	c.143dupT	p.Ser49Lysfs*2	Pathogenic	SCV000710113.1	PMID:25707398
**Chr21:38845176 -38845179**	NM_001347721.2	c.197_200delTAAC	p.Asn68fs*16	Pathogenic	VCV000598757	
**Chr21:38850536**	NM_001396.3	c.261delC	p.Pro88Glnfs*6	Pathogenic	-	PMID:28053047
**Chr21:38850565 -38850566**	NM_001396.4	c.290_291delCT	p.Ser97Cysfs*2	Pathogenic	VCV000418949.2	PMID:23099646
**Chr21:38850573**	NM_001396.3	c.298delT	p.Leu100*	Pathogenic	-	PMID:28053047
**Chr21:38850577**	NM_001396.3	c.302delT	p.Leu101*	Pathogenic	-	PMID:28053047
**Chr21:38850572 -38850576**	NM_101395.2	c.297_301delCTTGA	p.Leu100Glnfs*7	Pathogenic	SCV000485020.1	
**Chr21:38850586**	NM_001396.4	c.311dupA	p.Tyr104*	Pathogenic	SCV000651267.1	
**Chr21:38850587**	NM_001347721.2	c.285C>G	p.Tyr95*	Pathogenic	SCV000206785.1	PMID:25944381
**Chr21:38852961**	NM_001396.4	c.349C>T	p.Arg117*	Pathogenic	SCV000594471.1	
**Chr21:38852973**	NM_001347721.2	c.334C>T	p.Gln112*	Pathogenic	VCV000620014	
**Chr21:38853064**	NM_001396.4	c.452dupA	p.Asn151Lysfs*12	Pathogenic	SCV000206790.1	PMID:25944381
**Chr21:38853073**	NM_001396.3	c.461delA	p.Lys154Serfs*11	Pathogenic	SCV000206788.1	PMID:25944381

**Chr21:38853088**	NM_001347721.2	c.449dupA	p.Tyr150*	Pathogenic	SCV000484418.2	
**Chr21:38858786**	NM_001396.4	c.534dupT	p.Arg179Serfs*10	Pathogenic	SCV000829066.1	PMID:28492532
**Chr21:38858821 -38858824**	NM_001396.4	c.569_572delTAAA	p.Ile190Argfs*7	Pathogenic	SCV000708617.1	
**Chr21:38858865**	NM_001396.3	c.613C>T	p.Arg205*	Pathogenic	SCV000206786.1<br>SCV000807303.1	PMID:25944381<br>PMID:25326635
**Chr21:38858873 -38858876**	NM_001347721.2	c.594_597delinsGAA	p.Glu199fs*3	Pathogenic	VCV000162154	
**Chr21:38858918**	NM_001396.4	c.664+2T>C	splice donor variant	Pathogenic	SCV000820081.1	PMID:25944381<br>PMID:28492532
**Chr21:38862468 -38862472**	NM_001396.4	c.665-9_665-5delCTCTT	intron variant	Pathogenic	SCV000677027.1	PMID:25920557<br>PMID:25707398
**Chr21:38862503**	NM_001396.4	c.691C>T	p.Arg231*	Pathogenic	SCV000827498.1	PMID:28053047
**Chr21:38862575**	NM_001396.3	c.763C>T	p.Arg255*	Pathogenic	SCV000594473.1<br>SCV000833797.1	PMID:29034068<br>PMID:28053047<br>PMID:25920557<br>PMID:28492532
**Chr21:38862599**	NM_001396.3	c.787C>T	p.Arg263*	Pathogenic	SCV000329641.5	PMID:28053047
**Chr21:38862655**	NM_001396.3	c.621_624delinsGAA	p.Glu208Asnfs*3	Pathogenic	SCV000196059.1	PMID:25920557
**Chr21:38862656**	NM_001396.4	c.844dupA	p.Ser282Lysfs*6	Pathogenic	SCV000196064.1	PMID:25920557
**Chr21:38862672**	NM_001396.4	c.860A>T	p.Asp287Val	Pathogenic	VCV000437414	
**Chr21:38862744**	NM_001396.4	c.932C>T	p.Ser311Phe	Pathogenic	SCV000586742.1	PMID:28708303
**Chr21:38862749**	NM_001396.4	c.937C>T	p.Gln313*	Pathogenic	SCV000803733.1	
**Chr21:38862757**	NM_001396.4	c.945dupG	p.Gln316Alafs*24	Pathogenic	SCV000196061.1	PMID:25920557
**Chr21:38862767**	NM_001396.4	c.951+4_951+7	intron variant	Pathogenic	SCV000594475.1	PMID:27241786

**-38862770**		delAGTA			SCV000660433.2	PMID:26922654 PMID:28492532
**Chr21:38865403**	NM_001396.3	c.1036T>C	p.Ser346Pro	Pathogenic	SCV000196060.1	PMID:25920557
**Chr21:38865463**	NM_001347721.2	c.1069G>T	p.Glu357*	Pathogenic	VCV000664970	
**Chr21:38865466**	NM_001396.3	c.1098+1G>A	intron variant	Pathogenic	SCV000056591.2	PMID:23160955 PMID:25707398
**Chr21:38868422 -38868425**	NM_001396.3	c.1101_1104del AGAT	p.Asp368Argfs*2	Pathogenic	SCV000206789.1	PMID:25944381
**Chr21:38868483**	NM_001396.4	c.1162dupG	p.Ala388Glyfs*9	Pathogenic	SCV000245478.1	PMID:26633545
**Chr21:38868553**	NM_001396.4	c.1232dupG	p.Arg413Thrfs*10	Pathogenic	SCV000196066.1	PMID:25920557
**Chr21:38877644 -38877645**	NM_001396.4	c.1298_1299ins T	p.Pro434Thrfs*15	Pathogenic	SCV000247231.1	
**Chr21:38877655**	NM_001396.3	c.1309C>T	p.Arg437*	Pathogenic	SCV000247232.1 SCV000807305.1 SCV000822250.1	PMID:25326635 PMID:28053047 PMID:25920557 PMID:28492532
**Chr21:38877745**	NM_001347721.2	c.1372C>T	p.Arg458*	Pathogenic	VCV000204005	PMID:25944381 PMID:26633545
**chr21:38877746**	NM_001347721.2	c.1373G>A	p.Arg458Gln	Pathogenic	VCV000209150	PMID:28053047
**Chr21:38877837**	NM_001396.4	c.1491delC	p.Ala498Profs*94	Pathogenic	SCV000494645.1	PMID:23160955
**Chr21:38878494**	NM_001396.3	c.1639C>T	p.Gln547*	Pathogenic	SCV000265603.3	
**Chr21:38858815**	NM_001396.3	c.563A>T	p.Lys188Ile	Likely pathogenic	SCV000206791.1	PMID:25944381
**Chr21:38862546**	NM_001396.3	c.734T>G	p.Leu245Arg	Likely pathogenic	SCV000206792.1	PMID:25944381

**Chr21:38862641**	NM_001396.3	c.829G>C	p.Ala277Pro	Likely pathogenic	SCV000571206.3	PMID:28053047
**Chr21:38862695**	NM_001396.3	c.883C>T	p.Leu295Phe	Likely pathogenic	SCV000206793.1	PMID:25944381
**Chr21:38862734**	NM_001396.3	c.922T>G	p.Phe308Val	Likely pathogenic	SCV000247240.1	
**Chr21:38865339**	NM_001347721.2	c.945T>A	p.Ser315Arg	Likely pathogenic	VCV000627624	
**Chr21:38865347**	NM_001347721.2	c.953A>G	p.Tyr318Cys	Likely pathogenic	SCV000599256.1	
**Chr21:38865403**	NM_001347721.2	c.1009T>C	p.Ser337Pro	Likely pathogenic	VCV000162155	
**Chr21:38865404**	NM_001396.4	c.1037C>T	p.Ser346Phe	Likely pathogenic	SCV000712522.1	PMID:25533962 PMID:25920557
**Chr21:38877584**	NM_001347721.2	c.1213-2A>G	intron variant	Likely pathogenic	VCV000666564	
**Chr21:38877746**	NM_001396.3	c.1400G>A	p.Arg467Gln	Likely pathogenic	SCV000245477.1	PMID:26633545
**Chr21:38877751**	NM_001396.4	c.1405delC	p.Gln469Asnfs*123	Likely pathogenic	SCV000594477.1	
**Chr21:38884305**	NM_001396.3	c.1763C>A	p.Thr588Asn	Likely pathogenic	SCV000196065.1	
**Chr21:38853137**	NM_001396.3	c.516+9A>G	intron variant	Benign	SCV000660400.2	PMID:25707398
**Chr21:38862643**	NM_001396.4	c.831G>A	p.Ala277Ala	Benign	SCV000660401.2	
**Chr21:38865433**	NM_001396.4	c.1066A>G	p.Thr356Ala	Benign	SCV000651250.1	
**Chr21:38877579**	NM_001396.4	c.1240-7C>T	intron variant	Benign	SCV000651252.2	PMID:25707398

**Chr21:38877762**	NM_001396.4	c.1416T>C	p.Tyr472Tyr	Benign	SCV000651254.2
**Chr21:38878421**	NM_001396.4	c.1566C>T	p.Ser522Ser	Benign	SCV000651256.1
**Chr21:38884577**	NM_001396.3	c.2035G>C	p.Ala679Pro	Benign	SCV000651259.2
**Chr21:38844977**	NM_001396.4	c.11-9T>C	intron variant	Likely benign	SCV000651251.1
**Chr21:38845113**	NM_001396.4	c.138A>T	p.Pro46Pro	Likely benign	SCV000771155.1
**Chr21:38850536**	NM_001347721.2	c.234C>T	p.Asp78Asp	Likely benign	VCV000382661
**Chr21:38852966**	NM_001396.4	c.354A>G	p.Arg118Arg	Likely benign	SCV000771161.1
**Chr21:38852972**	NM_001396.4	c.360A>G	p.Gln120Gln	Likely benign	SCV000771158.1
**Chr21:38852987**	NM_001396.4	c.375C>T	p.Asp125Asp	Likely benign	SCV000651268.1
**Chr21:38865405**	NM_001396.4	c.1038C>G	p.Ser346Ser	Likely benign	SCV000651249.1
**Chr21:38868526**	NM_001396.4	c.1205C>G	p.Thr402Ser	Likely benign	SCV000771162.1
**Chr21:38877684**	NM_001396.4	c.1338C>T	p.Val446Val	Likely benign	SCV000651253.2
**Chr21:38878457**	NM_001396.4	c.1602G>A	p.Gln534Gln	Likely benign	SCV000771163.1
**Chr21:38878490**	NM_001396.4	c.1635C>T	p.Ala545Ala	Likely benign	SCV000771164.1
**Chr21:38884207**	NM_001396.4	c.1672-7C>T	intron variant	Likely	SCV000771160.1

				benign		
**Chr21:38884304**	NM_001396.4	c.1762A>C	p.Thr588Pro	Likely benign	SCV000771159.1	
**Chr21:38884336**	NM_001396.4	c.1794G>A	p.Leu598Leu	Likely benign	SCV000771157.1	
**Chr21:38884369**	NM_001396.4	c.1827T>C	p.His609His	Likely benign	SCV000651257.1	
**Chr21:38884387**	NM_001396.4	c.1845T>C	p.His615His	Likely benign	SCV000651258.1	
**Chr21:38884618**	NM_001396.4	c.2076T>C	p.Phe692Phe	Likely benign	SCV000651260.1	
**Chr21:38884654**	NM_001396.4	c.2112C>G	p.Val704Val	Likely benign	SCV000651261.1	
**Chr21:38884714**	NM_001396.4	c.2172T>C	p.Ala724Ala	Likely benign	SCV000651262.1	
**Chr21:38845022**	NM_001396.4	c.47G>A	p.Arg16Gln	Uncertain significance	SCV000651269.1	
**Chr21:38845102**	NM_001347721.2	c.127C>T	p.Arg43Cys	Uncertain significance	VCV000430291	
**Chr21:38845103**	NM_001347721.2	c.128G>A	p.Arg43His	Uncertain significance	VCV000659251	
**Chr21:38845114**	NM_001396.4	c.139A>G	p.Asn47Asp	Uncertain significance	VCV000581720	PMID:28492532
**Chr21:38845144**	NM_001347721.2	c.169T>C	p.Ser57Pro	Uncertain significance	VCV000651500	

**Chr21:38850482**	NM_101395.2	c.208-28G>A	intron variant	Uncertain significance	VCV000218379	PMID:26677511
**Chr21:38850514**	NM_001396.4	c.239G>T	p.Arg80Leu	Uncertain significance	SCV000822849.1	PMID:28492532
**Chr21:38850529**	NM_001347721.2	c.227T>G	p.Phe76Cys	Uncertain significance	VCV000656015	
**Chr21:38853043**	NM_001347721.2	c.404A>G	p.Asn135Ser	Uncertain significance	VCV000665902	
**Chr21:38853123**	NM_001347721.2	c.484G>A	p.Gly162Arg	Uncertain significance	VCV000655610	
**Chr21:38862474**	NM_001396.4	c.665-3C>T	intron variant	Uncertain significance	SCV000771156.1	
**Chr21:38862657**	NM_001396.4	c.845G>A	p.Ser282Asn	Uncertain significance	SCV000828531.1	PMID:28492532
**Chr21:38877588**	NM_001396.4	c.1242G>C	p.Glu414Asp	Uncertain significance	SCV000825823.1	
**Chr21:38877602**	NM_001347721.2	c.1229G>C	p.Gly410Ala	Uncertain significance	VCV000660992	
**Chr21:38877633**	NM_001396.4	c.1287G>A	p.Val429Val	Uncertain significance	VCV000383232	
**Chr21:38877803**	NM_001396.4	c.1457G>A	p.Gly486Asp	Uncertain significance	VCV000523623	
**Chr21:38877883**	NM_001396.4	c.1537T>A	p.Ser513Thr	Uncertain significance	SCV000651255.1	
**Chr21:38878409**	NM_001347721.2	c.1527A>G	p.Ser509Ser	Uncertain significance	VCV000625936	

**Chr21:38878442**	NM_001396.4	c.1587G>A	p.Ser529Ser	Uncertain significance	SCV000771149.1	
**Chr21:38878450**	NM_001396.4	c.1595C>T	p.Thr532Met	Uncertain significance	SCV000771153.1	
**Chr21:38878465**	NM_001396.4	c.1610delA	p.His537fs*55	Uncertain significance	VCV000641967	
**Chr21:38878476**	NM_001396.4	c.1621C>T	p.His541Tyr	Uncertain significance	SCV000771151.1	
**Chr21:38878512**	NM_001396.4	c.1657A>G	p.Thr553Ala	Uncertain significance	SCV000771150.1	
**Chr21:38884254**	NM_001347721.2	c.1685C>T	p.Thr562Ile	Uncertain significance	VCV000662754	
**Chr21:38884271**	NM_001396.4	c.1729G>C	p.Val577Leu	Uncertain significance	SCV000771148.2	PMID:28492532
**Chr21:38884274**	NM_001347721.2	c.1705A>G	p.Thr569Ala	Uncertain significance	VCV000585805	
**Chr21:38884281**	NM_001347721.2	c.1712A>G	p.Glu571Gly	Uncertain significance	VCV000657128	
**Chr21:38884331**	NM_001396.4	c.1789G>A	p.Ala597Thr	Uncertain significance	SCV000813203.1	PMID:28492532
**Chr21:38884381 -38884386**	NM_001396.4	c.1839_1844dup CCA	p.His609_His610d up	Uncertain significance	SCV000814041.1	PMID:28492532
**Chr21:38884384 -38884386**	NM_001396.4	c.1842_1844dup CCA	p.His610dup	Uncertain significance	SCV000771152.1	
**Chr21:38884443**	NM_001347721.2	c.1874A>G	p.Asn625Ser	Uncertain significance	VCV000287612	

**Chr21:38884567**	NM_001396.4	c.2025A>C	p.Gln675His	Uncertain significance	SCV000771154.1	
**Chr21:38884662**	NM_001396.4	c.2120A>G	p.Asn707Ser	Uncertain significance	SCV000771147.1	
**Chr21:38884667**	NM_001347721.2	c.2098C>T	p.Arg700Cys	Uncertain significance	VCV000641739	
**Chr21:38884719**	NM_001396.4	c.2177C>G	p.Thr726Arg	Uncertain significance	SCV000651263.2	PMID:28492532
**Chr21:38884785**	NM_001396.4	c.2243C>T	p.Ser748Phe	Uncertain significance	SCV000651264.1	
**Chr21:38884793**	NM_001396.4	c.2251A>T	p.Thr751Ser	Uncertain significance	SCV000651265.1	

Notes: del, deletion; *, nonsense variant; fs, frameshift variant.
